# Relapse predictability of topological signature on pretreatment planning CT images of stage I non‐small cell lung cancer patients before treatment with stereotactic ablative radiotherapy

**DOI:** 10.1111/1759-7714.14483

**Published:** 2022-06-16

**Authors:** Takumi Kodama, Hidetaka Arimura, Yuko Shirakawa, Kenta Ninomiya, Tadamasa Yoshitake, Yoshiyuki Shioyama

**Affiliations:** ^1^ Division of Medical Quantum Science, Department of Health Sciences, Graduate School of Medical Sciences Kyushu University Fukuoka Japan; ^2^ Division of Medical Quantum Science, Department of Health Sciences, Faculty of Medical Sciences Kyushu University Fukuoka Japan; ^3^ National Hospital Organization Kyushu Cancer Center Fukuoka Japan; ^4^ Sanford Burnham Prebys Medical Discovery Institute La Jolla California USA; ^5^ Department of Clinical Radiology, Graduate School of Medical Sciences Kyushu University Fukuoka Japan; ^6^ Ion Beam Therapy Center, SAGA HIMAT Foundation Tosu Japan

**Keywords:** non‐small cell lung cancer (NSCLC), radiotherapy, relapse, topology

## Abstract

**Background:**

This study aimed to explore the predictability of topological signatures linked to the locoregional relapse (LRR) and distant metastasis (DM) on pretreatment planning computed tomography images of stage I non‐small cell lung cancer (NSCLC) patients before treatment with stereotactic ablative radiotherapy (SABR).

**Methods:**

We divided 125 primary stage I NSCLC patients (LRR: 34, DM: 22) into training (*n* = 60) and test datasets (*n* = 65), and the training dataset was augmented to 260 cases using a synthetic minority oversampling technique. The relapse predictabilities of the conventional wavelet‐based features (WF), topology‐based features [BF, Betti number (BN) map features; iBF, inverted BN map features], and their combined features (BWF, iBWF) were compared. The patients were stratified into high‐risk and low‐risk groups using the medians of the radiomics scores in the training dataset.

**Results:**

For the LRR in the test, the iBF, iBWF, and WF showed statistically significant differences (*p* < 0.05), and the highest nLPC was obtained for the iBF. For the DM in the test, the iBWF showed a significant difference and the highest nLPC.

**Conclusion:**

The iBF indicated the potential of improving the LRR and DM prediction of stage I NSCLC patients prior to undergoing SABR.

## Graphical abstract

We investigated the predictability of topological signatures on planning CT images linked to locoregional relapse (LRR) and distant metastasis (DM) of non‐small cell lung cancer (NSCLC) patients after stereotactic ablative radiotherapy (SABR). A signature derived from inverted Betti number map features indicated the potential of improving the high‐risk and low‐risk stratification for LRR and DM of stage I NSCLC patients prior to undergoing SABR.

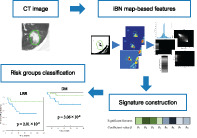

## INTRODUCTION

Lung cancer is one of the most common type of cancers worldwide, with almost 2.2 million new cases (11.4%) being identified in 2020 and 1.8 million new deaths (18.0%) occurring in the same year.^1^ The two main types of lung cancer are small cell lung cancer and non‐small cell lung cancer (NSCLC), with NSCLC accounting for approximately 85% of all lung cancer cases.^2^ At present, surgery is the first treatment option for patients with stage I NSCLC, and stereotactic ablative radiotherapy (SABR) is recommended for inoperable patients.^3^ However, the outcomes of stage I NSCLC patients, who received SABR, have been found to be potentially comparable with those of surgery,^4^, ^5^, ^6^, ^7^, ^8^ whereas there have been reports of patients with locoregional relapses (LRRs) and distant metastases (DMs) after SABR.^5^, ^8^

Effective adjuvant therapies are needed for SABR patients at the highest risk of relapse to reduce the risk of developing DM.^9^ Hence, the pretreatment prediction of patient prognosis, especially cancer relapse, is crucial for the choice more appropriate treatment options. The use of radiomics may predict cancer relapse and/or distant metastasis in patients with early‐stage NSCLC.^10^, ^11^ In conventional radiomics, intratumor intensity heterogeneity has been quantified using histogram and texture analyses,^10^, ^11^ since recurrent lung tumors tend to exhibit ground‐glass opacities (GGO) surrounding the consolidative changes.^12^ Additionally, we focused on not only the intratumor intensity heterogeneity, but also on small blobs (connected components), holes, or cavitations in the binary images with intratumoral regions on the computed tomography (CT) images. In particular, the prognostic power of the presence of a cavitation in lung cancer has been investigated in previous studies.^13^, ^14^, ^15^ Topology can quantify the intensity heterogeneity, holes, and cavitations in lung cancer by calculating the radiomics features that predict prognosis in lung cancer patients.^16^, ^17^ Betti numbers (BNs) in topology represent topological invariants that indicate the underlying property of the objects under continuous deformation. The two types of BNs in a two‐dimensional image may be defined as zero‐ and one‐dimensional BNs, representing the number of connected components (B0) and the number of holes (B1), respectively.^18^, ^19^ Ninomiya et al.^17^ newly developed a BN map and demonstrated that the prognostic power of BN map‐based features to characterize intratumor heterogeneity was superior to that of conventional features and deep learning techniques.

We hypothesized that signatures based on the topology‐based features obtained from pretreatment planning CT images could stratify stage I NSCLC patients into high‐ and low‐risk groups with respect to cancer relapse after the use of SABR. This study aimed to explore the predictability of topological signatures linked with time to the LRR and DM in stage I NSCLC patients treated with SABR to determine whether the use of SABR is appropriate for each patient. Furthermore, this study introduced two types of BN maps (original BN map and inverted BN map), which were obtained from binary images and inverted binary images, respectively, because the original and inverted BN maps may take advantage of topological connectivity within the tumor and background regions.

## METHODS

### Clinical cases

This retrospective study was approved by the Institutional Review board of the Kyushu University Hospital. We employed a pretreatment planning CT dataset of NSCLC patients (*n* = 125; *n*: number of patients) treated with SABR at our university hospital. Table 1 summarizes the clinical information of the patients. The SABR‐treated patients comprised 80 inoperable cases because of old age, impaired pulmonary function, and comorbidities of the cancer, 38 surgery refusal cases, and seven unknown cases. The treatment protocols were 40–54 Gy/4 Fr (*n* = 121) and 60–70 Gy/10 Fr (*n* = 4). The entire dataset (*n* = 125) was divided into training (*n* = 65) and test (*n* = 60) datasets to balance the proportion of the number of events and censored cases for LRR and DM in each dataset. There were no statistically significant differences (*p* > 0.05; Mann–Whitney U test) in the distributions of the times to the LRR and DM in cases in the two datasets.

**TABLE 1:** Summary of clinical information

	NSCLC patients (*n* = 125)
Age (year, min–max [median])	60–91 (78)
Time to LRR (month, min–max [median])	3–162 (29)
Time to DM (month, min–max [median])	3–162 (32)
Effective diameter of tumor (mm, min–max [median])	10–53 (27)
Sex	
Male	90
Female	35
Stage	
IA	77
IB	48
Prognosis	
LRR	34
DM	22
Relapse free	81
Component	
Solid	96
Part‐solid	19
GGO	10
Histopathology	
Adenocarcinoma	73
Squamous cell carcinoma	43
Large cell carcinoma	4
Unknown	5

Abbreviations: DM, distant metastasis; GGO, ground‐glass opacity; LRR, locoregional relapse.

**FIGURE 1:**
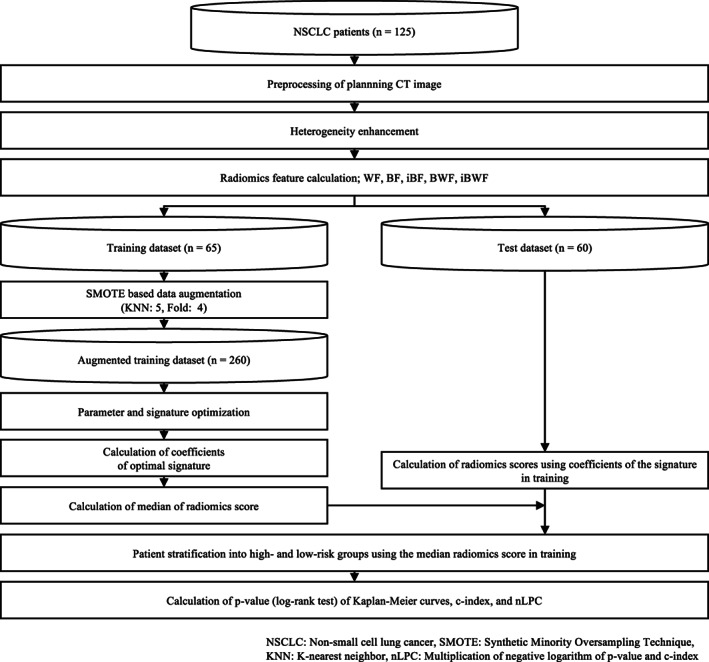
Overall workflow of radiomics prediction used in this study

The planning CT images were axial slice images with a matrix size of 512 × 512; a pixel size of 0.78, 0.84, 0.88, or 0.98 mm; and a slice thickness of 2 or 3.2 mm. The anisotropic CT images and gross tumor volumes (GTVs) that were used for the treatment were transformed into isotropic voxel images (iso‐voxel size: 0.98 mm) using cubic and shape‐based interpolation,^20^ respectively. Prior to the main step (heterogeneity enhancements in Figure 1), two preprocessing techniques, requantization and edge enhancement, were applied to the isotropic voxel CT images. Requantized 8 bits images were generated based on a look‐up table ranging from 0 to 255, which corresponded to a range window of Hounsfield units (HUs). The range of the HUs was optimized in a parameter optimization. A LoG filter was applied to the requantized images to enhance the lung cancer patterns and reduce the noise.

### Overall workflow

Figure 1 shows the overall workflow of this study. Heterogeneity within tumors of CT images was enhanced using original and inverted BN mappings based on topology and conventional three‐dimensional (3D) wavelet decomposition. Radiomics features were extracted from the original CT and heterogeneity enhanced images analyzed using histogram and texture analysis. A wavelet‐based feature (WF), original BN map feature (BF), and inverted BN map feature (iBF) were calculated as radiomics features.  An augmented training dataset (*n* = 260) was generated from the training dataset using a synthetic minority oversampling technique (SMOTE)‐based method, which enabled the overfitting problem to be avoided. The hyperparameters and a signature were optimized based on a robustness index (RI), and a radiomics score was calculated from the signature. The signature was constructed from an optimal set of significant features that were selected by a Cox‐net algorithm. Patients were divided into high‐ and low‐risk groups with respect to the medians of the radiomics scores, and the proposed approach was assessed using the *p*‐value obtained for the Kaplan–Meier (KM) curves of the two groups, the c‐index, and the multiplication of the negative logarithm of the *p‐*value and the c‐index (nLPC), which is considered to be a comprehensive evaluation index. The calculations of the preprocessing and the radiomics features were used in a MATLAB 2019a environment with a MATLAB‐based radiomics tool package^21^, ^22^ and in‐house programs. The calculations of the Cox‐net algorithm and evaluations computing *p*‐values of the log‐rank test, c‐indices, and nLPCs were used in the R‐4.0.4 environment.^23^, ^24^, ^25^

### Heterogeneity enhancement and feature calculation

**FIGURE 2:**
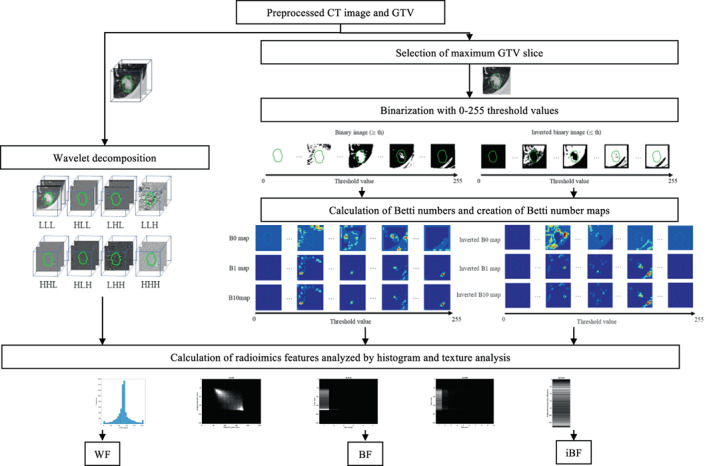
Heterogeneity enhancement using 3D wavelet decomposition and original Betti number (BN) and inversed BN mapping

Figure 2 showed a workflow of heterogeneity enhancement using 3D wavelet decomposition and original BN and inverted BN mapping. The BF, iBF, and WF were calculated from the radiomics‐enhanced images. The BF was calculated from the BN maps which were analyzed using histogram and texture analysis. The BN maps were created from images binalized with 0–255 threshold values on an axial slice image with a maximum GTV region of the preprocessed CT images, as mentioned in the last subsection.

**FIGURE 3:**
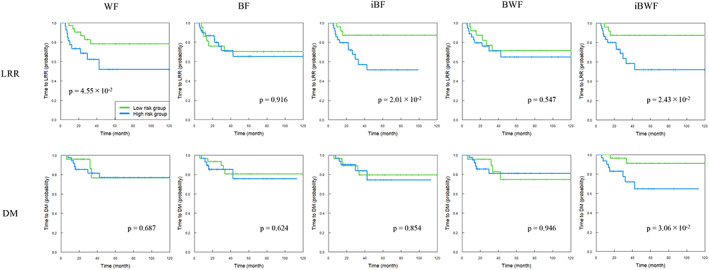
Kaplan–Meier curves of time to locoregional relapse (LRR) and distant metastasis (DM) for test dataset based on radiomics scores derived from five signatures based on a wavelet‐based feature (WF), original Betti number (BN) map feature (BF), inverted BN map feature (iBF), BF and WF combined feature (BWF), and iBF and WF combined feature (iBWF). Patients were stratified into high‐ and low‐risk groups by the median radiomics score of the training dataset

In this study, two types of BN maps were introduced. These were obtained from binary images as well as inverted binary images. The connected component of B0 was defined as connected pixels based on eight neighbors, whereas the hole of the B1 was defined based on four neighbors. The BNs were calculated from binary images of the threshold CT images, as mentioned previously. The B0 and B1 regions represented one (e.g., tumor) region based on the eight‐neighbor connectivity and the zero (e.g., background) region based on the four‐neighbor connectivity. The inverted BN maps were calculated from inverted binary images that were generated as 0 for pixels with a value greater than the threshold and one for pixels with a value smaller than the threshold. For example, the B0 and B1 derived from the inverted binary images may have represented the background regions based on the eight‐neighbor connectivity; however, the tumor regions were based on the four‐neighbor connection. Therefore, the original and inverted BN maps could characterize the different properties of the tumor and background regions.

The iBF was obtained by combining the BF and the features calculated from the inverted BN maps. The inverted BN maps were calculated from the inverted binary images. The inverted BN calculation and mapping for the same as that for the original BN. A total of 82 998 features were computed for the iBF. The details of the feature calculation are described in the supplement file (Data S1).

The conventional WF was calculated from the original CT and 3D wavelet decomposed images. The preprocessed CT images were decomposed into eight filtered images using low‐pass (L) or high‐pass (H) wavelet filters (i.e., LLL, HLL, LHL, LLH, HHL, HLH, LHH, and HHH filters) in the x‐, y‐, and z‐directions.^26^ The scaling filter was the Coiflet scaling function (“coif1”). The WF was derived from nine images: the original CT image and eight wavelet decomposition images. Finally, a total of 486 features were calculated from the nine types of images according to a histogram and texture analysis.

The radiomics features (BF, iBF, WF) were normalized to a mean of zero and a standard deviation of one before the data augmentation and signature construction.

### SMOTE‐based data augmentation

The training dataset (*n* = 65) was augmented to 260 patients using the SMOTE‐based method before the parameters and signature optimization. The augmentation method was used to quadruple the number of patients in the augmented datasets compared to the original training dataset. SMOTE is a data augmentation method based on the k‐nearest neighbors algorithm.^27^ The details of the data augmentation are described in the supplement file (Data S2).

### Hyperparameters and signature optimization

Hyperparameters and signature optimization were performed in the augmented dataset. The augmented training dataset (*n* = 260) was divided randomly into a training subdataset (*n* = 130) and a validation subdataset (*n* = 130) for the optimization. The eight bits requantization range 𝑅 of HUs, kernel size *K*, shift pixel 𝑆 in the calculation of BNs, and elastic net blending parameter α in the Cox‐net regularization of the signature construction were optimized, and an optimal signature was constructed with the best significant features set decided using the maximum RI. The RI was calculated from nLPCs of the training and validation subdatasets, and it could find the most robust parameters and signature set between the training and validation. The details of RI calculation were described in the supplement file (Data S3). For the training subdataset, a signature was constructed with a set of all possible combination of seven significant features. The significant features were decided in the order of the most frequently selected features that corresponded to the Cox‐net nonzero coefficients. Patients in both the training and validation subdatasets were stratified into high‐ and low‐risk groups using the median radiomics score of the training subdataset. The radiomics scores were calculated using the sum of multiplication of the signature and the coefficients based on the Cox proportional hazard model (CPHM) for the training subdatasets. For the validation subdatasets, the radiomics scores were calculated using the same coefficients of the training subdatasets, in the same way. The details of the parameter and signature optimization are described in the supplement file (Figure S1).

### Combined features

Two types of combined features for BF and WF (BWF) or iBF and WF (iBWF) were investigated. These combined features were generated from the features with optimal heterogeneity enhancement parameters ($\hat{R},\hat{K},\hat{S}$). The blending parameter α and the signature combination were optimized as was done for the other features.

### Evaluation of relapse predictability of the optimal signature

The relapse predictabilities for the training and validation subdatasets were assessed using the *p*‐value in the KM analysis, c‐index, and comprehensive evaluation index, which was the multiplication of the negative logarithm of the *p*‐value and c‐index (nLPC). The *p*‐values were evaluated using a log‐rank test in the KM curves for times to the LRR and DM between the high‐risk and low‐risk groups, and a *p* < 0.05 was considered to indicate a statistically significant difference between the two risk groups. Harrell's c‐index (0–1) was calculated in the training and test datasets to evaluate the predictive performance of the radiomics score for the times to the LRR and DM.^28^, ^29^ A c‐index of 1 indicated perfect prediction and a c‐index of 0.5 indicated a random guess. The c‐index was calculated from a response score that was defined as a minus radiomics score. The nLPC was considered to be a comprehensive evaluation index. A larger nLPC value indicated better prognostic predictability.

The relapse predictability of the optimal signature that was derived from the radiomics features was verified through an analysis with the test dataset. The patients in the test dataset were stratified into high‐ and low‐risk groups using the median radiomics score in the training dataset, and the relapse predictability of each feature was evaluated with *p‐*values (log‐rank test) of KM curves, c‐index, and nLPC.

## RESULTS

Table 2 shows the optimal parameters and signature. The optimal CT window width $\hat{R}$ was set as −1350 to 150 (lung range) for all the features for the LRR and DM. The optimal kernel size and shift pixel were nine and three, respectively, for the BF and iBF of the LRR and DM.

**TABLE 2:** Optimal parameters (a) and signature (b)

(a)					
	Requantization range (HU)	Kernel size	Shift pixel	Regularization term (*α*)	No. of features in signature
LRR					
WF	−1350 to 150	–	–	0.3	5
BF	−1350 to 150	9	3	0.4	4
iBF	−1350 to 150	9	3	0.5	3
BWF	–	–	–	0.5	3
iBWF	–	–	–	1.0	5
DM					
WF	−1350 to 150	–	–	0.7	5
BF	−1350 to 150	9	3	0.8	5
iBF	−1350 to 150	9	3	0.9	5
BWF	–	–	–	0.9	5
iBWF	–	–	–	1.0	5

(b)					
	Signature features				
LRR					
WF	GLCM_Correlation_HHL	Histogram_Skewness_HLL	Histogram_Minimum_LLH	GLSZM_GLV_LLH	Histogram_Median_LHH
BF	GLRLM_SRLGE_B1_th237	GLSZM_SZHGE_B10_th74	GLSZM_ZP_B0_th165	GLCM_AutoCorrelation_B0_th240	–
iBF	Histogram_Energy_iB0_th235	GLRLM_SRLGE_B1_th237	GLRLM_RLV_B1_th106	–	–
BWF	GLSZM_SZLGE_B1_th215	GLRLM_RLV_B1_th106	GLSZM_LZE_LLL	–	–
iBWF	GLSZM_SZLGE_B1_th198	GLSZM_LZE_LLL	GLRLM_SRLGE_B1_th237	GLSZM_SZHGE_B10_th74	GLRLM_RLV_iB10_th177
DM					
WF	Histogram_Skewness_HLL	Histogram_Energy_HLH	GLCM_Correlation_LHL	GLSZM_GLN_HLL	NGTDM_Contrast_Org
BF	GLSZM_SZLGE_B10_th67	GLSZM_SZLGE_B10_th233	GLSZM_SZE_B1_th76	GLSZM_SZLGE_B0_th111	Histogram_Entropy_B10_th235
iBF	GLSZM_SZLGE_iB10_th234	GLSZM_SZLGE_iB1_th136	GLSZM_SZE_iB0_th126	GLRLM_SRHGE_iB10_th153	GLSZM_ZSV_B0_th245
BWF	Histogram_Skewness_HLL	GLSZM_SZLGE_B10_th67	GLSZM_SZE_B1_th76	GLSZM_SZLGE_B10_th95	GLSZM_SZLGE_B1_th221
iBWF	Histogram_MeanAbsoluteDeviation_iB0_th121	Histogram_Skewness_HLL	GLSZM_SZLGE_iB1_th136	GLSZM_SZLGE_iB10_th113	GLSZM_LZE_iB10_th226

Abbreviations: WF, wavelet‐based feature; BF, original BN map feature; BWF, BF and WF combined feature; iBF, inverted BN map feature; iBWF, iBF and WF combined feature; LRR, locoregional relapse; DM, distant metastasis; th, threshold value.

For the LRR for the test dataset (Table S2), the *p‐*values (log‐rank test) of KM curves were 4.55 × 10^−2^ for the WF, 0.916 for the BF, 2.01 × 10^−2^ for the iBF, 0.547 for the BWF, and 2.43 × 10^−2^ for the iBWF. For the DM for the test dataset (Table S2), the *p*‐values were 0.687 for the WF, 0.624 for the BF, 0.854 for the iBF, 0.946 for the BWF, and 3.06 × 10^−2^ for the iBWF. Figure 3 shows the KM curves for the times to the LRR and DM for the high‐ and low‐risk patient groups of the test dataset.

**FIGURE 4:**
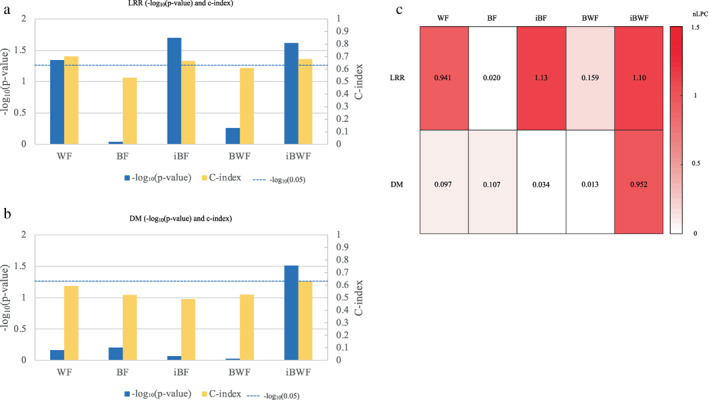
Bar plots of negative logarithm of *p*‐values (log‐rank test) to the base ten and c‐indices for locoregional relapse (LRR) (a) and distant metastasis (DM) (b) in the test dataset, and a heat map of nLPCs obtained from the test (c)

The c‐indices for the LRR for the test dataset were 0.701 for the WF, 0.532 for the BF, 0.665 for the iBF, 0.608 for the BWF, and 0.680 for the iBWF (Table S2). The c‐indices for the DM for the test dataset were 0.594 for the WF, 0.522 for the BF, 0.488 for the iBF, 0.524 for the BWF, and 0.628 for the iBWF (Table S2).

The nLPCs for the WF, BF, iBF, BWF, and iBWF were 0.941, 2.03 × 10^−2^, 1.13, 0.159, and 1.10 for the LRR for the test dataset, respectively, and 9.68 × 10^−2^, 0.107, 3.35 × 10^−2^, 1.26 × 10^−2^, and 0.952 for the DM for the test dataset, respectively (Table S2).

Figure 4 shows the comparison of the evaluation indices obtained from the radiomics features using bar plots of the negative logarithm of the *p*‐values (log‐rank test) with the base ten and c‐indices for the LRR and DM in the test dataset and a heat map of the nLPCs obtained from the test. The iBF for the LRR and iBWF for the DM showed the lowest *p‐*values and the highest nLPCs in the test dataset.

**FIGURE 5:**
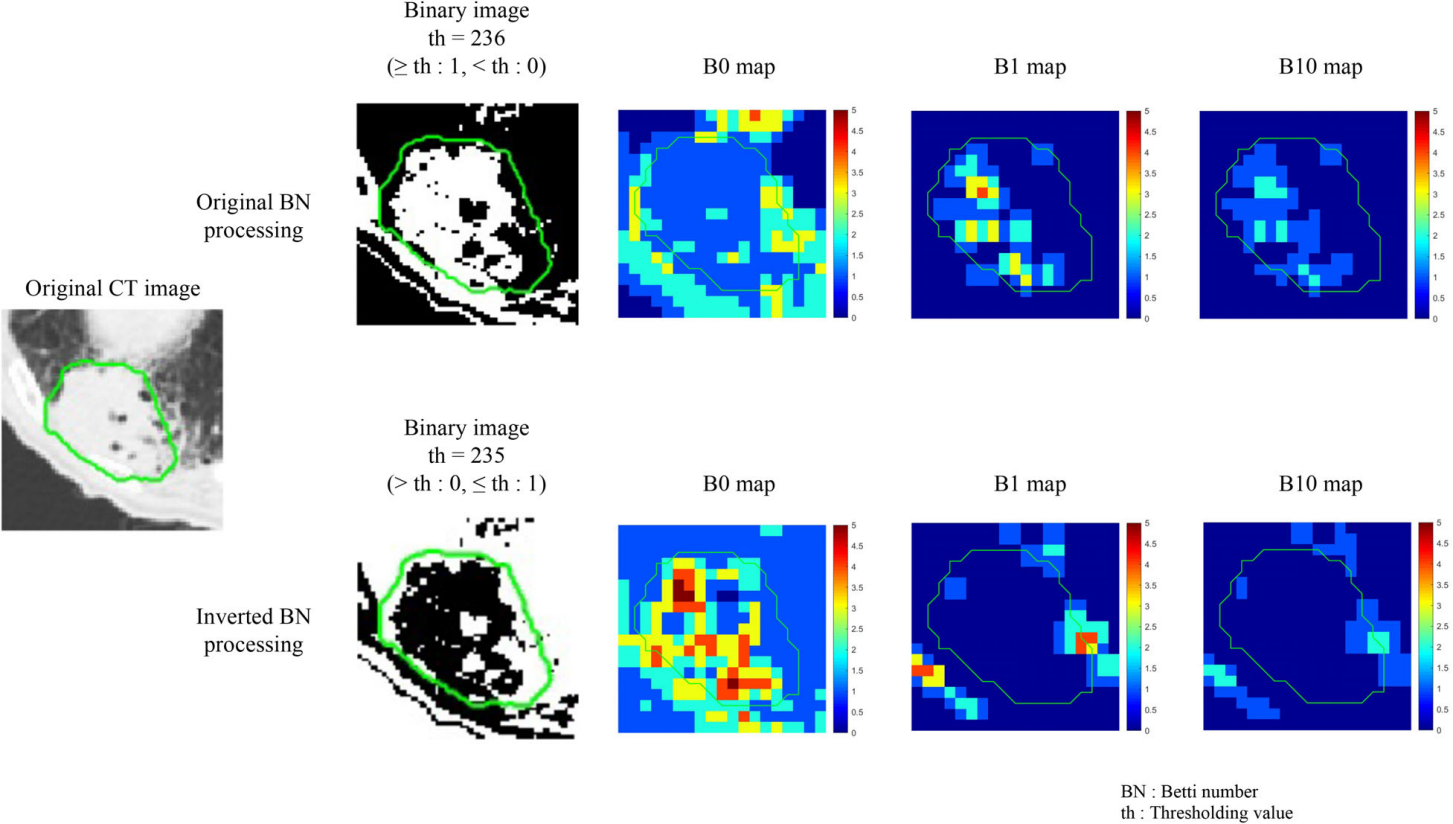
Comparison of binary images and Betti maps for original Betti number (BN) map feature (BF) (upper) and inverted BN map feature (iBF) (lower) in a patient

Figure 5 shows comparison of the binary images and their BN maps for the BF and the iBF. Both the inverted B0 map and original B1 map represented the BNs for the tumor region, and the inverted B0 (iB0) map showed a higher distribution than the original B1 map. An inverted significant feature (“Histogram_Energy_iB0 _th235”; Table 2) obtained from the inverted B0 map was selected for the iBF for the LRR.

**FIGURE 6:**
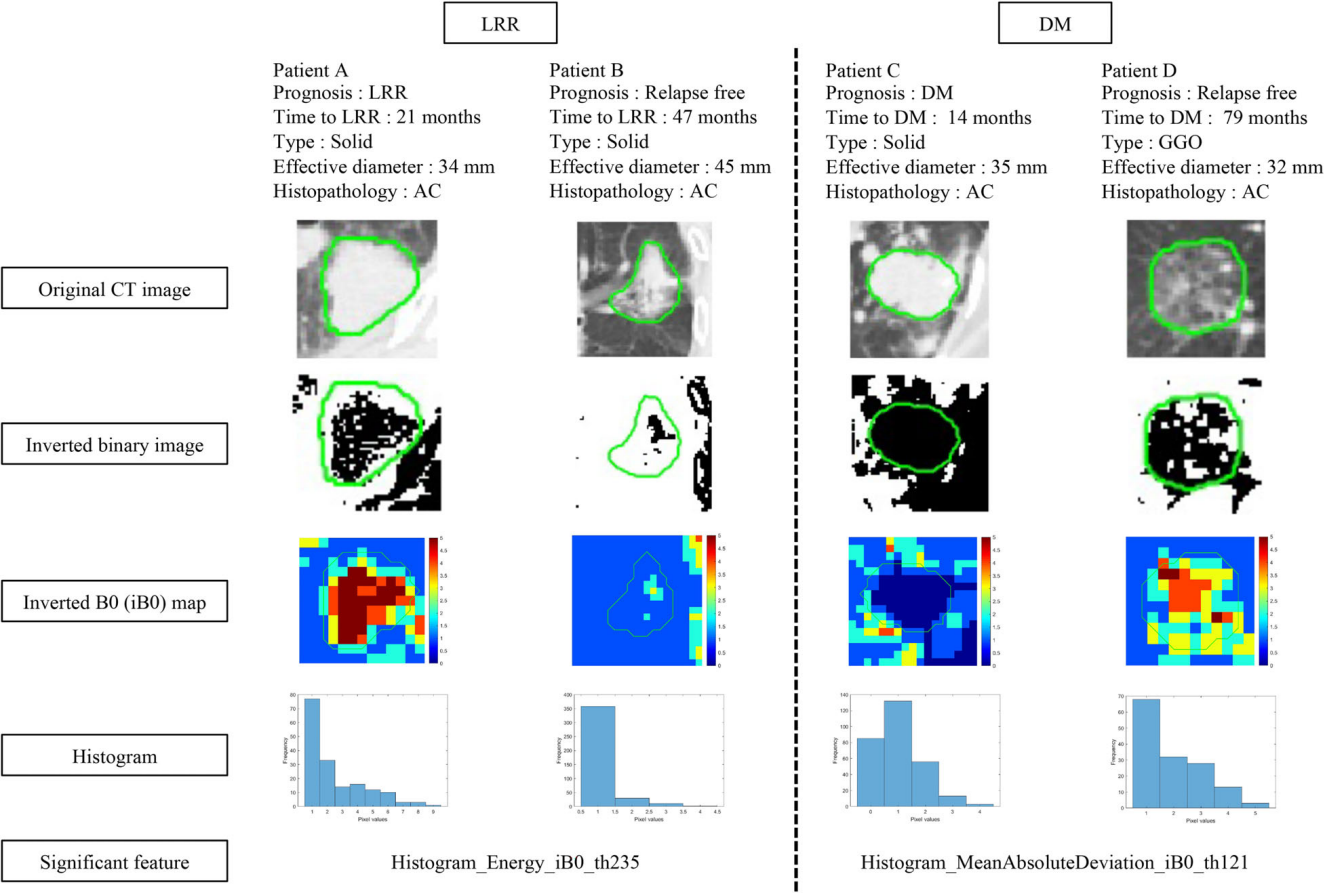
Images of patients with relapse and relapse free linked to significant features with the largest Cox‐net coefficient of inverted Betti number (BN) map feature (iBF) for locoregional relapse (LRR) and iBF and wavelet‐based feature combined feature (iBWF) for distant metastasis (DM). AC: Adenocarcinoma.

Figure 6 shows the patients with the images linked to a significant feature with the highest coefficient of the iBF for the LRR and the iBWF for the DM.

## DISCUSSION

Figure 5 shows a comparison of the original and inverted BN maps in the computation of the significant BF and iBF for the LRR in a patient with an original CT image and with threshold CT images. The inverted B0 map generated from the inverted binary image representing the cavity as pixel value one may be comparable with the original B1 map. In the figure, the inverted B0 map showed higher BN distributions than the original B1 map. A previous study^17^ indicated that the size of a detectable cavity in the original B1 maps depended on the size of the kernel, because the original B1 numbers were calculated in the kernel and the B1 holes had to be included completely. The inverted B0 map may have detected cavities larger than the kernel size because it counted B0 connected components even though they were included partially in the kernel.

Figure 6 shows a comparison of the patients with relapse and the relapse free patients for the LRR and DM using images for the computation of significant features with the largest coefficient. The LRR for Patients A and B showed different patterns for the inverted B0 maps which represented distributions of the background regions. The feature “Energy” was calculated using the summation of the squares of pixel values in the inverted B0 map, and it represented a distribution of pixels with large values. Therefore, a higher inverted B0 number distribution (larger number of cavities in the tumor) at a threshold of 235 (≃1383HU) may be a significant prognostic feature for the LRR. For the DM, Patient D represented a heterogeneous pattern in the inverted B0 map at a threshold of 121 (≃715HU). The feature “MeanAbsoluteDeviation” refers to an absolute deviation from a mean, and it represents a statistical variability of the inverted B0 numbers. The tumor type of Patient D was GGO, and the GGO was shown to indicates a better prognosis for the DM.^30^ The inverted B0 map at a threshold of 121 may extract significant prognostic features such as GGO.

Some previous studies have demonstrated radiomics prediction models for cancer relapse and metastasis in patients with early‐stage NSCLC.^10^, ^11^ Kakino et al.^10^ predicted a local relapse and DM using the radiomics features on breath‐hold CT images of early‐stage NSCLC patients. They analyzed wavelet‐based radiomics features and the features combined with clinical features using a random survival forest model, and they demonstrated statistically significant differences of cumulative incidence curves (*p* = 0.021; Gray's test) of the high‐ and low‐risk groups of the DM for the features. Wu et al.^11^ predicted the DM in patients with NSCLC using a quantitative radiomics approach involving the extraction of imaging features from pretreatment fluorodeoxyglucose (FDG) positron emission tomography (PET) images. They showed *p*‐values (log‐rank test) of 4.98 × 10^−2^ of the KM curves derived from a PET quantitative imaging radiomics feature (PF)‐based model and 2.89 × 10^−2^ for a combined PF and histological type feature (PH)‐based model. The c‐indices for PFs and PHs were 0.71 and 0.80, respectively. The nLPC was calculated as 0.92 and 1.23 for PFs and PHs, respectively. The proposed method obtained the significant differences for the KM curves of the LRR of the two risk groups derived from a conventional WF, and iBF and iBWF. The CPHM based radiomics score may show better LRR predictability, and the inverted BN map features may enhance the predictability. The iBWF also may demonstrate the significant differences for the stratification of the risk groups, comparable to the previous study.

For the LRR in the test, the iBF, iBWF, and WF showed statistically significant differences (*p* < 0.05), and the highest nLPC was obtained by the iBF. For the DM in the test, the iBWF showed a significant difference and the highest nLPC. The iBF indicated the potential for improving the LRR and DM prediction of stage I NSCLC patients prior to the use of SABR. The iBF‐based signatures can predict the applicability of the SABR in stage I NSCLC patients. Timmerman et al.^31^ mentioned that the use of SABR in operable patients may be compared ideally in a phase 3 randomized clinical trial in USA, and some clinical studies to perform such a trial are ongoing. If clinical trials on the use of SABR for operable patients are performed in Japan, the radiomic signatures for prediction of the applicability of the use of SABR or surgery may be explored.

There were three limitations to this study. First, we enrolled a relatively small number of patients (*n* = 125) in this study. A small dataset may lead to lower generalizability and affect the robustness of the signature. The present method used a SMOTE‐based data augmentation to ensure a larger dataset. In future studies, the number of patients should be increased. Second, the impact of the CT imaging protocols and the manufacturers on the predictability was not investigated in this study. The vulnerability of radiomics features extracted from CT images for the images acquisition and reconstruction algorithms was noted in previous studies.^32^, ^33^ The last limitation was that only one axial slice image with a maximum tumor region for each patient was used in the BN calculation. We used the maximum region slice because it was too time‐consuming to obtain the BN maps from the whole slices of the tumor. Even though we demonstrated the superior performance of 2D topological features compared with 3D WFs, we recommend investigating whether 3D topological features can provide improved prognostic predictability.

In conclusion, we investigated the relapse predictability of the iBF derived from the pretreatment planning CT images of early‐stage NSCLC patients undergoing SABR. The iBF has shown the potential to characterize new tumor properties associated with the risk of LRR on the CT images more accurately than the conventional radiomics features. In addition, it was found that the iBF showed greater predictability for the DM when they were combined with the WF.

## CONFLICT OF INTEREST

The authors declare that they have no relevant or material financial interests that relate to the research described in this study.

## Supporting information


**Appendix S1** Supporting information
