# Supplementary material for: Relapse predictability of topological signature on pretreatment planning CT images of stage I non‐small cell lung cancer patients before treatment with stereotactic ablative radiotherapy
Source: Thorac Cancer. 2022 Jun 16;13(15):2117–26. doi: 10.1111/1759-7714.14483 (PMC9346172; doi:10.1111/1759-7714.14483)
Supplement: Supplementary file 1 — Appendix S1 Supporting information [file TCA-13-2117-s001.docx]

Radiomics features

Significant features selection by 100 times iteration of Cox-net

$\left( \hat{R},\hat{KS},\hat{SP},\hat{\alpha},\hat{SF} \right)=\underset{R,KS, SP,\alpha,SF}{argmax} RI$

Random split

Augmented training dataset (n = 260)

Training sub dataset (n = 130)

Validation sub dataset (n = 130)

Signature construction

Calculation of median of radiomics scores

Calculation of radiomics scores using

coefficients of the signature in training

Patient stratification into high- and low-risk groups using the median radiomics score in training

Calculation of p-value (log-rank test) of Kaplan-Meier curves, c-index, and nLPC

Calculation of coefficients and

radiomics scores of signature

Calculation of robustness index (RI) of nLPCs in training and validation

Number of significant features: $m$ = 7

Signature $SF$construction with all possible combinations of significant features

Number of combinations

$=\sum_{l=1}^{m} {}_{m}{C_{l}}$

**FIGURE S1.** Workflow of parameters and signature optimization. 8 bits re-quantization range 𝑅 (-1000 to 1500 HU, -150 to 250 HU [mediastinal range], -1350 to 150 HU [lung range]) and blending parameter 𝛼 (0-1; interval: 0.1) were optimized for WF, Kernel size 𝐾 (5, 7, 9), shift pixel 𝑆 (1, 2, 3), 𝑅, and 𝛼 were optimized for BF and iBF, and 𝛼 was optimized for BWF and iBWF.

**TABLE S1.** Radiomics features calculation methods

| Histogram-based features (14) | Texture features (40) | | | |
| --- | --- | --- | --- | --- |
|  | GLCM (9) | GLRLM (13) | GLSZM (13) | NGTDM (5) |
| Energy | Energy | Short run emphasis (SRE) | Small zone emphasis (SZE) | Coarseness |
| Entropy | Contrast | Long run emphasis (LRE) | Large zone emphasis (LZE) | Contrast |
| Kurtosis | Entropy | Gray level non-uniformity (GLN) | Gray level non-uniformity (GLN) | Busyness |
| Maximum | Homogeneity | Run length non-uniformity (RLN) | Zone-size non-uniformity (ZSN) | Complexity |
| Mean | Correlation | Run percentage (RP) | Zone percentage (ZP) | Strength |
| Mean absolute difference | Variance | Low gray level run emphasis (LGRE) | Low gray level zone emphasis (LGZE) |  |
| Median | Sum average | High gray level run emphasis (HGRE) | High gray level zone emphasis (HGZE) |  |
| Minimum | Dissimilarity | Short run low gray level emphasis (SRLGE) | Small zone low gray level emphasis (SZLGE) |  |
| Range | Auto correlation | Short run high gray level emphasis (SRHGE) | Small zone high gray level emphasis (SZHGE) |  |
| Root mean square |  | Long run low gray level emphasis (LRLGE) | Large zone low gray level emphasis (LZLGE) |  |
| Skewness |  | Long run high gray level emphasis (LRHGE) | Large zone high gray level emphasis (LZHGE) |  |
| Standard deviation (SD) |  | Gray level variance (GLV) | Gray level variance (GLV) |  |
| Uniformity |  | Run-length variance (RLV) | Zone-size variance (ZSV) |  |
| Variance |  |  |  |  |
| GLCM: Gray-level co-occurrence matrix, GLRLM: Gray-level run-length matrix, GLSZM: Gray-level size-zone matrix, NGTDM: Neighbor gray-tone difference matrices | | | | |

**TABLE S2.** LRR and DM predictabilities of 5 signatures based on WF, BF, iBF, BWF, and iBWF

|  |  |  | P-value (HR, 95%CI) | C-index (95%CI) | nLPC | RI |
| --- | --- | --- | --- | --- | --- | --- |
| Training subdataset  (n = 130) | LRR | WF | 1.81 × 10^-11^ (0.02, 0.01 - 0.04) | 0.906 (0.86 - 0.95) | 9.74 | - |
|  |  | BF | 9.53 × 10^-12^ (0.02, 0.01 - 0.04) | 0.893 (0.85 - 0.94) | 9.84 | - |
|  |  | iBF | 1.55 × 10^-13^ (0.02, 0.01 - 0.04) | 0.935 (0.89 - 0.98) | 12.0 | - |
|  |  | BWF | 1.02 × 10^-10^ (0.08, 0.04 - 0.15) | 0.924 (0.90 - 0.95) | 9.23 | - |
|  |  | iBWF | 1.28 × 10^-13^ (0.02, 0.01 - 0.04) | 0.946 (0.92 - 0.97) | 12.2 | - |
|  | DM | WF | 1.03 × 10^-9^ (0, 0 - 0) | 0.916 (0.88 - 0.95) | 8.14 | - |
|  |  | BF | 8.18 × 10^-10^ (0, 0 - 0) | 0.931 (0.89 - 0.97) | 8.46 | - |
|  |  | iBF | 3.06 × 10^-10^ (0, 0 - 0) | 0.925 (0.89 - 0.96) | 8.80 | - |
|  |  | BWF | 3.15 × 10^-10^ (0, 0 - 0) | 0.913 (0.87 - 0.96) | 8.68 | - |
|  |  | iBWF | 3.58× 10^-10^ (0.02, 0.01 - 0.06) | 0.957 (0.94 - 0.98) | 9.04 | - |
| Validation subdataset  (n = 130) | LRR | WF | 3.04 × 10^-12^ (0.04, 0.02 - 0.07) | 0.848 (0.79 - 0.91) | 9.76 | 19.0 |
|  |  | BF | 4.14 × 10^-12^ (0.02, 0.01 - 0.04) | 0.867 (0.81 - 0.93) | 9.87 | 19.2 |
|  |  | iBF | 4.52 × 10^-14^ (0.02, 0.01 - 0.03) | 0.895 (0.83 - 0.96) | 11.9 | 23.1 |
|  |  | BWF | 3.23 × 10^-11^ (0.09, 0.04 - 0.17) | 0.883 (0.83 - 0.94) | 9.27 | 17.8 |
|  |  | iBWF | 6.72 × 10^-14^ (0, 0 - 0) | 0.925 (0.89 - 0.96) | 12.2 | 24.1 |
|  | DM | WF | 8.85 × 10^-10^ (0, 0 - 0) | 0.906 (0.86 - 0.95) | 8.20 | 15.4 |
|  |  | BF | 2.69 × 10^-10^ (0.02, 0.01 - 0.06) | 0.878 (0.80 - 0.96) | 8.40 | 15.9 |
|  |  | iBF | 1.35 × 10^-10^ (0, 0 - 0) | 0.905 (0.85 - 0.96) | 8.94 | 15.6 |
|  |  | BWF | 1.94 × 10^-10^ (0.02, 0.01 - 0.05) | 0.895 (0.85 - 0.94) | 8.69 | 17.1 |
|  |  | iBWF | 2.24 × 10^-10^ (0.02, 0.01 - 0.05) | 0.939 (0.91 - 0.97) | 9.06 | 17.7 |
| Test dataset  (n = 60) | LRR | WF | 4.55 × 10^-2^ (0.37, 0.14 - 1.02) | 0.701 (0.58 - 0.82) | 0.941 | - |
|  |  | BF | 0.916 (0.95, 0.35 - 2.59) | 0.532 (0.40 - 0.67) | 2.03 × 10^-2^ | - |
|  |  | iBF | 2.01 × 10^-2^ (0.25, 0.10 - 0.68) | 0.665 (0.53 - 0.80) | 1.13 | - |
|  |  | BWF | 0.547 (0.73, 0.27 - 1.97) | 0.608 (0.47 - 0.74) | 0.159 | - |
|  |  | iBWF | 2.43 × 10^-2^ (0.26, 0.10 - 0.70) | 0.680 (0.53 - 0.83) | 1.10 | - |
|  | DM | WF | 0.687 (0.76, 0.21 - 2.76) | 0.594 (0.41 - 0.78) | 9.68 × 10^-2^ | - |
|  |  | BF | 0.624 (0.74, 0.21 - 2.58) | 0.522 (0.32 - 0.72) | 0.107 | - |
|  |  | iBF | 0.854 (0.89, 0.26 - 3.08) | 0.488 (0.35 - 0.63) | 3.35 × 10^-2^ | - |
|  |  | BWF | 0.946 (0.96, 0.27 - 3.37) | 0.524 (0.36 - 0.69) | 1.26 × 10^-2^ | - |
|  |  | iBWF | 3.06 × 10^-2^ (0.21, 0.10 - 0.68) | 0.628 (0.49 - 0.76) | 0.952 | - |
|  |  |  | HR: Hazard ratio, 95%CI: 95% confidential interval, RI: Robustness index | | | |

Table S2 shows the p-values (log-rank test) of Kaplan-Meier curves, c-indices, nLPCs, and RIs for LRR and DM of each dataset. For the training and validation subdataset, the evaluation indices and RI calculated from the optimal parameters and signatures were shown.

**DOCUMENT S1.** Radiomics features calculation

BF was calculated from BN maps generated by counting BNs on thresholded binary images of original images. The binary images were generated as 1 for pixels with a value greater than the threshold and 0 for pixels with a value smaller than the threshold. The maximum GTV slice image was selected from the 3D preprocessed CT image. Three types of BN maps were produced from BNs in local regions of interest determined from kernel sizes 𝐾, shift pixels 𝑆, and dimensions of BNs. BNs were computed from the 256 binary images by counting the number of connected components (B0) and holes (B1) and the B1/B0 ratio (B10). The kernel sizes and shifting pixels used in the calculation of BN maps determined the sizes of detectable holes and sampling intervals of BNs, respectively^1^. Finally, a total of 41,526 features were calculated from the original image and BN map according to 54 equations of histogram and texture features. The texture features of the equations were derived from gray-level co-occurrence matrices (GLCM)^2^, gray-level run-length matrices (GLRLM)^3^, gray-level size-zone matrices (GLSZM)^4^, and neighbor gray-tone difference matrices (NGTDM)^5^. The radiomics features of the 54 calculation methods were calculated from GTVs used for SABR. The 54 methods were listed in Table S1. iBF and WF also calculated by the 54 calculation methods.

**DOCUMENT S2.** Synthetic minority oversampling technique-based method (SMOTE) -based data augmentation

The training dataset (n = 65) was augmented to 260 patients using the SMOTE-based method before parameters and signature optimization. The augmentation method was computed to quadruple the number of patients in the augmented datasets to the training dataset. SMOTE is a data augmentation method based on *k* nearest neighbor algorithm^6^. First, for each reference patient’s feature vector in the training dataset, *k* nearest neighbor patients were found, and *l* (number of selected patients) patients was selected randomly in the *k* neighbors. Then, new patients’ feature vectors were generated between the reference and selected patients’ vectors with a random ratio. In this study, number of the *k* and *l* were set to five and three, and the three new patients were generated to quadruple the number of patients in augmented training dataset. Times to relapse (LRR and DM) of the new patients were calculated from the reference and selected patients’ times to relapse using the ratio used to generate the feature vector. We did not adjust the ratio of the number of LRR or DM cases and that of censored cases, assuming that the proposed method would be practiced with clinical data.

**DOCUMENT S3.** Calculation of robustness index (RI) in parameter and signature optimization

The optimal parameters and signatures were selected by maximizing the robustness index (RI)^7^. RI was calculated from nLPCs of the training and validation sub datasets to evaluate the robustness of nLPCs obtained from parameters and signature sets in the training and validation. The RI was calculated as follows:

$$\begin{aligned} RI=\frac{{nLPC}_{t}+{nLPC}_{v}}{1+\left| {nLPC}_{t}-{nLPC}_{v} \right|}\#\left( 1 \right) \end{aligned}$$

where ${nLPC}_{t}$ and ${nLPC}_{v}$ are the nLPCs calculated in the training and validation sub datasets.

**REFERENCES**

1 Ninomiya K, Arimura H. Homological radiomics analysis for prognostic prediction in lung cancer patients. *Phys Medica*. 2020; **69**: 90-100. doi:10.1016/j.ejmp.2019.11.026.

2 Haralick RM, Dinstein I, Shanmugam K. Textural features for image classification. *IEEE Trans Syst Man Cybern.* 1973. doi: https://doi.org/10.1109/TSMC.1973.4309314.

3 Galloway MM. Texture analysis using gray level run lengths. *Comput Graph Image Process* 1975; **4**: 172–9. doi: https://doi.org/10.1016/S0146-664X(75)80008-6.

4 Thibault G, Fertil B, Navarro C, et al. Texture indexes and gray level size zone

matrix application to cell nuclei classification. *Pattern Recognit Inf Process* 2009.

5 Amadasun M, King R. Textural features corresponding to textural properties. *IEEE Trans Syst Man Cybern.* 1989; **19**: 1264–74. doi: <https://doi.org/10.1109/21.44046>.

6 Chawla, Nitesh V, Bowyer K et al. SMOTE: synthetic minority over-sampling technique. *J Artif Intell Res*. 2002; **16**: 321-357.

7 Ninomiya K, Arimura H, Chan WY, et al. Robust radiogenomics approach to the identification of EGFR mutations among patients with NSCLC from three different countries using topologically invariant Betti numbers. *PLoS One*. 2021; **16**: 1-17. doi: 10.1371/journal.pone.0244354.
